# Methylation Patterns of the *HTR2A* Associate With Relapse-Related Behaviors in Cocaine-Dependent Participants

**DOI:** 10.3389/fpsyt.2020.00532

**Published:** 2020-06-10

**Authors:** Michelle A. Land, Divya Ramesh, Aaron L. Miller, Richard B. Pyles, Kathryn A. Cunningham, F. Gerard Moeller, Noelle C. Anastasio

**Affiliations:** ^1^Center for Addiction Research, Department of Pharmacology and Toxicology, University of Texas Medical Branch, Galveston, TX, United States; ^2^Department of Psychiatry and Institute for Drug and Alcohol Studies, Virginia Commonwealth University, Richmond, VA, United States; ^3^Department of Pediatrics, University of Texas Medical Branch, Galveston, TX, United States

**Keywords:** cocaine use disorder, *HTR2A*, methylation, impulsivity, attentional bias, rs6311, A-1438G

## Abstract

Relapse during abstinence in cocaine use disorder (CUD) is often hastened by high impulsivity (predisposition toward rapid unplanned reactions to stimuli without regard to negative consequences) and high cue reactivity (e.g., attentional bias towards drug reward stimuli). A deeper understanding of the degree to which individual biological differences predict or promote problematic behaviors may afford opportunities for clinical refinement and optimization of CUD diagnostics and/or therapies. Preclinical evidence implicates serotonin (5-HT) neurotransmission through the 5-HT_2A_ receptor (5-HT_2A_R) as a driver of individual differences in these relapse-related behaviors. Regulation of 5-HT_2A_R function occurs through many mechanisms, including DNA methylation of the *HTR2A* gene, an epigenetic modification linked with the memory of gene-environment interactions. In the present study, we tested the hypothesis that methylation of the *HTR2A* may associate with relapse-related behavioral vulnerability in cocaine-dependent participants versus healthy controls. Impulsivity was assessed by self-report (Barratt Impulsiveness Scale; BIS-11) and the delay discounting task, while levels of cue reactivity were determined by performance in the cocaine-word Stroop task. Genomic DNA was extracted from lymphocytes and the bisulfite-treated DNA was subjected to pyrosequencing to determine degree of methylation at four cytosine residues of the *HTR2A* promoter (-1439, -1420, -1224, -253). We found that the percent methylation at site -1224 after correction for age trended towards a positive correlation with total BIS-11 scores in cocaine users, but not healthy controls. Percent methylation at site -1420 negatively correlated with rates of delay discounting in healthy controls, but not cocaine users. Lastly, the percent methylation at site -253 positively correlated with attentional bias toward cocaine-associated cues. DNA methylation at these cytosine residues of the *HTR2A* promoter may be differentially associated with impulsivity or cocaine-associated environmental cues. Taken together, these data suggest that methylation of the *HTR2A* may contribute to individual differences in relapse-related behaviors in CUD.

## Introduction

Cocaine use disorder (CUD) continues to be a significant public health problem, with rising cocaine-related overdose deaths linked to the growing opioid crisis (1). Cocaine use begins against a background of genetics and environment, although the intricate interplay between these variables is still poorly understood and differs between individuals, presenting a barrier to understanding the origins of CUD as well as moving forward toward efficacious pharmacotherapeutics. Motor impulsivity (behavioral disinhibition) and impulsive choice (decision-making) are two dimensions of impulsivity that associate with CUD relapse-associated behaviors (2–5). Impulsivity is interlocked with cue reactivity (6, 7) which is defined as the attentional orientation toward drug-associated stimuli that predict reward (8, 9). Cocaine-dependent subjects often present with high levels of impulsivity (3, 4, 10) and cue reactivity (11–13). Furthermore, high levels of impulsivity are negatively correlated with treatment retention in cocaine-dependent individuals (10, 14). At present, CUD is a chronic relapsing disorder with no known Food and Drug Administration-approved medications.

Gene expression and function are regulated through genetic and epigenetic mechanisms essential for cellular differentiation, function, and development (15–17). DNA methylation is one of several types of epigenetic modifications which is essential in imprinting chromosomal DNA with the memory of past gene-environment interactions (15, 18). At the core of epigenetics is a series of proteins that establish DNA methylation and histone modification patterns (writers), those that interpret these patterns by selective binding (readers), and those that erase the patterns (erasers) during epigenetic reprogramming (19). Methylation occurs at CpG islands, which are stretches of DNA with a higher concentration of cytosines and guanines (18). These CpG islands are located in the promoter region of genes and DNA methylation at CpG sites generally results in inhibited gene transcription through obscured transcription factor binding sites (18, 20). Up to 70–80% of cytosines in CpG islands are subjected to methylation and altered epigenetic profiles are associated with a wide range of neuropsychiatric diseases (21, 22), including substance use disorders (23).

Studying the influence of epigenetic differences provides an opportunity to greatly improve diagnosis and treatment outcome by identifying interindividual responses to facilitate the development of precision pharmacotherapies. Epigenetics is a vital point of regulation necessary to fine tune transcriptional and translational processes such that the cell and ultimately the organism adapts to its environment. There is mounting evidence that serotonin (5-HT) neurotransmission through the 5-HT_2A_ receptor (5-HT_2A_R) controls neural mechanisms underlying relapse-related behavioral vulnerability to cocaine, including both impulsivity and cue reactivity (for reviews) (6, 7). In preclinical models, selective 5-HT_2A_R antagonists administered systemically consistently reduce impulsivity and cue-primed drug seeking (24–29). In particular, pharmacological and genetic association studies implicate 5-HT_2A_R regulation of impulsive choice (30–33). Further, methylation of the *HTR2A* (human 5-HT_2A_R gene) is implicated as a contributor to schizophrenia and mood disorders (34–36), but has not been interrogated in impulsivity or CUD relapse-related behaviors. Here, we profiled the methylation pattern of the *HTR2A* promoter from blood lymphocyte DNA collected from healthy controls and cocaine-dependent participants. Tracking epigenetic changes in the blood provides a useful clinical tool as gene targets in the periphery, like the brain, are subjected to an array of changes following exposure to environmental stimuli. We tested the hypothesis that the DNA methylation profile of the *HTR2A* promotor from peripheral lymphocytes aligns to individual differences in impulsivity and cocaine cue reactivity in healthy controls and cocaine-dependent participants.

## Materials and Methods

### Participants

The sample consisted of 48 healthy controls and 53 cocaine-dependent participants recruited from ongoing studies at the University of Texas Health Science Center at Houston (n=10 healthy controls; n=10 cocaine users) or at the Institute for Drug and Alcohol Studies at Virginia Commonwealth University (VCU) (n=38 healthy controls; n=43 cocaine users) using the same diagnostic, psychometric, and advertising procedures. Participants were recruited *via* newspaper advertisements and were initially screened by a brief telephone interview. Individuals were excluded if they indicated significant psychiatric or medical conditions, including a self-reported history of severe brain injury. Following the phone screen, eligible participants attended an in-person intake assessment session, during which they completed a medical history and physical examination and were screened for psychiatric disorders using the structured clinical interview for DSM-IV (SCID-I) (37). Information about the participants' demographic and drug use history was also collected at the intake interview. All participants were urine tested for cocaine (benzoylecgonine), tetrahydrocannabinol (THC), opiates, amphetamine, methamphetamine, and benzodiazepines using integrated E–Z split key cup II (Innovacon Company, San Diego, CA) on each visit to the clinic.

Eligible participants met current DSM-IV criteria for cocaine dependence, did not meet DSM-IV current dependence criteria for drugs other than cocaine, marijuana, nicotine, or alcohol and did not have current or past medical disorders affecting the central nervous system. The cocaine-dependent sample included both treatment-seeking (n= 41) and non-treatment-seeking (n=12) participants. The treatment-seekers were part of studies in which they received manualized cognitive behavioral therapy and were randomized to either placebo or any one or combination of the following medications: levodopa/carbidopa and/or citalopram. All data from treatment-seekers were collected at intake prior to the start of medication or behavioral therapy; therefore treatment-seekers and non-treatment-seekers were grouped together for the analyses. The healthy control group consisted of participants who had a negative urine drug screen, negative breathalyzer test, and did not have any current or past DSM-IV axis I disorders (including any substance dependence) or medical disorders affecting the central nervous system. Healthy controls were recruited *via* similar advertising procedures as the cocaine-dependent participants. Healthy controls who were also smokers (n=13) were excluded from all analyses except for genotyping due to the influence of nicotine on baseline impulsivity task performance [(38–41); Moeller laboratory, unpublished observations].

All participants were free of alcohol at the time of testing as determined by a breathalyzer (Intoximeters, Inc., St. Louis, MO). Female participants were excluded if they had a positive urine pregnancy test. All participants were compensated for their participation. Participants were fully informed of the nature of the research and provided written consent for their involvement in accordance with the Declaration of Helsinki. The studies from which participant data were included were approved by the University of Texas Health Science Center at Houston, VCU, and University of Texas Medical Branch Institutional Review Boards.

### Barratt Impulsiveness Scale (BIS-11)

The BIS-11 is one of the most commonly used questionnaire-based measures of trait impulsivity (42). The BIS-11 is a 30-item self-report scale with three oblique second order factors: (1) attentional/cognitive impulsivity, measuring tolerance for cognitive complexity, and persistence; (2) motor impulsivity, measuring the tendency to act on the spur of the moment; and (3) non-planning impulsivity, measuring the lack of sense of the future. Items were rated from 1 (absent) to 4 (most extreme); total scores are a summation of attention, motor, and non-planning attributes, and ranged from 30–120, with non-psychiatric controls generally scoring 50–60. The BIS-11 questionnaire was completed by most participants except for two participants from the healthy controls and four participants from the cocaine users, these individuals were not included in the BIS-11 analyses.

### Adjusting Delay Discounting Task

The adjusting delay discounting task (43, 44) is designed to measure discounting rate when participants are presented with the possibility of receiving a hypothetical reward determined using a choice algorithm. The task (as previously described) (39) is presented on a computer screen displaying two large command buttons, one on the left and one on the right side of the screen, in which the choices are presented. The left button always displays an immediate adjusting reward, and the right button displays a delayed reward. Participants were exposed to a series of choices with varying reward magnitudes and delay periods during which indifference points between the adjusted immediate reward and the delayed reward were recorded for each set of delays. Participants were randomly assigned to complete the assessment in either ascending or descending order of delays. Choice presentations ended once indifference points were determined for each magnitude at each delay. The indifference points were the analyzed for each participant using a nonlinear regression and the following equation: *V* = 1/(*A*+k*D*) (44, 45), where *V* is the indifference point, *A* is the amount of the delay reward, and *D* is the delay. The result of the regression is the best-fitting k, this is a free parameter related to the rate of discounting. The natural logarithm of transformation, log10k, was used to normalize the distribution of k across participants for further statistical analyses. The adjusting delay discounting task was completed by most participants except for 14 healthy controls and 15 cocaine users; these individuals were excluded from the delay discounting analyses.

### Cocaine-Word Stroop Task

The cocaine-word Stroop task was designed to measure attentional bias to cocaine-related stimuli (38, 46–50). As previously described (38, 47, 48, 50), each analyzed session began with a block of 60 practice trials, two blocks of 30 trials with cocaine-related words, and two blocks of 30 trials with neutral words. Trials with correct responses and reaction times larger than 200 msec were used to calculate mean reaction times (38, 47, 48, 50). Attentional bias was operationalized as the difference between the reaction times (in msec) observed in trials with cocaine-related words and trials with neutral words, calculated for each subject and averaged across subjects (51). A correct response was defined as responding to the word color on an appropriately colored response button. Accuracy was assessed as the ratio of correct trials to total trials within each block type. The cocaine-word Stroop task was completed by healthy controls and most cocaine users except for five; these individuals were excluded from the attentional bias analyses.

### DNA Collection and Isolation

Venous blood (10 ml) from each subject was centrifuged at 2,000 rpm for 30 min (Eppendorf North America, Inc., NY). The buffy coat, containing lymphocytes and platelets, was removed, and stored in 2.0 ml cryogenic vials at -80°C. DNA was isolated from the buffy coat using the Puregene Kit (Qiagen Inc., CA) according to manufacturer's recommendations. Purified DNA for each subject was dissolved in 0.25 ml of DNA hydration solution. An aliquot of each DNA sample (50 µl) was transferred to a 96-well plate for pyrosequencing analysis by the Assay Development Service Division at the University of Texas Medical Branch.

### *HTR2A* Pyrosequencing

The methylation state of CpG sites within the *HTR2A* promoter was evaluated through bisulfite conversion of genomic DNA with subsequent pyrosequencing. Briefly, DNA was subjected to bisulfite conversion using an Epitect bisulfite kit (Qiagen) following the manufacturer's recommendations. CpG sites were then interrogated with a PCR – pyrosequencing approach (52). Bisulfite-converted DNA was used as template in PCR reactions consisting of 12.5 µl of PyroMark Master Mix containing coral load reagent (Qiagen) or iQ supermix^™^ (Bio-Rad, Hercules, CA, assay CpG 102) that was mixed within a 25 µL PCR reaction containing 200 nM of both biotinylated and standard primers, and nuclease-free water. Thermocycling was completed using a Bio-Rad C1000^™^ thermocycler. Generated biotinlyated PCR products were pyrosequenced and methylation state quantified using PyroMark Gold reagents on a PyroMark Q96 ID platform using CpG Software (Qiagen). Sequencing primer concentrations varied from 0.3–0.45 µM.

### *HTR2A* Genotyping

There is a single nucleotide polymorphism (SNP) of the *HTR2A* (rs6311; G A) at site -1438 that results in the loss of a CpG site (52, 53). Genotyping of site -1438 was accomplished using the same pyrosequencing workflow with the exceptions of bisulfite conversion and CpG software analysis. The forward primer (5'AAACACTGTTGGCTTTGGATGG3'), reverse primer (5' Biotin-TATGTCCTCRGAGTGCTGTGA3'), and sequencing primer (5'TTGGATGGAAGTGCC3') were designed in house. Polymorphism status was determined through PyroMark Q96 software. Individuals homozygous for the rs6311 SNP do not possess the -1439 CpG methylation site, therefore, homozygous individuals from the healthy controls (n=5) and cocaine users (n=8) were excluded from analyses of the -1439 site.

### Statistical Analysis

All statistical analysis were performed using GraphPad Prism software Version 7.02 or IBM^®^ SPSS^®^ Statistics package Version 1.0.0.1298. All individuals were pyrosequenced, however, for some individuals, the reaction was not possible for certain CpG sites; for these sites, these individuals were not included in the analysis. A Mann Whitney test was used to compare percent methylation between healthy controls and cocaine users. A two-way ANOVA with Sidak's multiple comparisons test was used to determine differences in the BIS-11 scores between healthy controls and cocaine users. A Students *t*-test was used to determine if delay discounting rates (log10k values), age and years of education were significantly different between healthy controls and cocaine users. A Friedman test with Dunn-Bonferroni multiple comparisons was carried out to compare the percent methylation of the four CpG sites within healthy controls or within cocaine users. A repeated measures ANOVA with Bonferroni multiple comparisons was performed to assess within subject differences for BIS-11 measures of healthy controls or cocaine users. All correlations were generated using the nonparametric Spearman's correlation. A nonparametric partial correlation was used for analyses corrected for age and years of cocaine use. A Chi-square test with Fisher's exact test was used to determine differences in sex, race, smoking, and marijuana use in healthy controls versus cocaine users. The Chi-square test with Fisher's exact test was performed to determine if allelic frequency of the rs6311 SNP was different between healthy controls and cocaine users. The alpha level for all analyses was set at *p*=0.05.

## Results

### Assessment of *HTR2A* Methylation Profile in Healthy Controls and Cocaine-Dependent Participants

**Figure 1:**
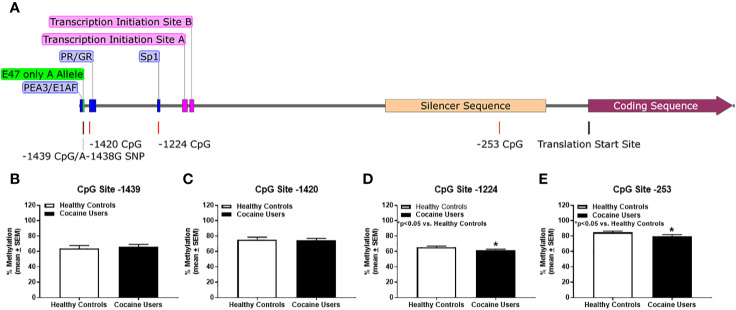
Cocaine users demonstrate hypomethylation of the *HTR2A* promoter versus healthy controls. **(A)** *HTR2A* gene is represented as the gray line and contains the promoter region, exon 1, exon 2, and the initial segment of the coding sequence of the gene. Transcription factor binding sites are represented in blue and green. CpG islands and SNPs are represented as red lines and transcription initiation sites are in pink. Translation start site is represented as a black line. The silencer sequence is annotated in tan and the coding sequence is in maroon. Comparison of percent methylation between healthy controls and cocaine users for **(B)** CpG site -1439, **(C)** -1420, **(D)** -1224 and **(E)** -253. *p < 0.05 versus healthy controls.

A careful analysis of the literature identified four key methylation sites within the *HTR2A* promoter that correspond to potential transcription factor binding sites: -1439, -1420, -1224, and -253 (**Figure 1A**). The first CpG site identified is located at -1439 (**Figure 1A**, maroon line); this is a binding site for the polyoma enhancer activator 3/early region 1A enhancer-binding protein (PEA3/E1AF) transcription factors (**Figure 1A**, blue box) (54–56). The rs6311 SNP in the *HTR2A* gene (**Figure 1A**, maroon line) at site -1438 removes the -1439 CpG methylation site and introduces an E47 transcription factor binding site (**Figure 1A**; green box) (52, 53). In carriers of the rs6311 SNP at site -1438, the guanine (G) is converted to an alanine (A), and hence the -1439 CpG island is missing (**Figure 1A**, maroon line). In both healthy controls and cocaine users, there was a mix of individuals who were heterozygous and homozygous for the rs6311 SNP. Using a Chi-squared analysis with Fisher's exact test, the allelic frequency of the rs6311 SNP (A-1438G) was not significantly different between cocaine users (G/G: n=21; A/G: n=24; A/A: n=8) versus healthy controls (G/G: n=18; A/G: n=25; A/A: n=5) (n.s.). Individuals heterozygous for the rs6311 SNP lose one of the CpG sites thereby reducing their maximum percent methylation to 50% as compared to homozygous for the wild type G allele at the -1439 CpG site. Individuals homozygous for the rs6311 SNP have no -1439 CpG site and were excluded from the percent methylation analyses for CpG site -1439. The next CpG site under study is at -1420 (**Figure 1A**, red line) to which the progesterone receptor (PR) and glucocorticoid receptor (GR) bind (52, 57) (**Figure 1A**, blue box). The -1224 CpG site (**Figure 1A**, red line) corresponds to the binding site of specificity protein 1 (Sp1) (**Figure 1A**, blue box) (52, 54). The final CpG site -253 (**Figure 1A**, red line) is in a known silencer region that spans from nucleotides -120 to -578 (**Figure 1A**, tan box) of the *HTR2A* promoter (54).

Demographic data for cocaine users and healthy controls were collected, including age, sex, race, years of education as well as drug use. The sex and race of healthy controls and cocaine users did not differ (n.s.; **Table 1**). Cocaine users were significantly older with fewer years of education and greater smoking and marijuana use (p < 0.05, **Table 1**). In healthy controls, age correlated significantly with percent methylation at site -1224 (R=-0.312, p < 0.05; **Table 2**) and -253 (R=-0.336, p < 0.05; **Table 2**), but not at CpG sites -1439 and -1420 (n.s.; **Table 2**). Age significantly correlated with percent methylation at site -1224 (R=-0.407, p < 0.05; **Table 2**), but was not significant at CpG sites -1439, -1420, and -253 (n.s.; **Table 2**) for cocaine users. Years of cocaine use significantly correlated with percent methylation at site -253 (R=-0.293, p < 0.05; **Table 2**), but was not significant at CpG sites -1439, -1420, and -1224. Years of smoking significantly correlated with percent methylation at site -1224 (R=-0.635, p < 0.05; **Table 2**), but not at CpG sites -1439, -1420, and -253 for cocaine users.

**Table 1:** Demographic analyses of healthy controls and cocaine users.

Characteristics	Healthy Controls (N=48)	Cocaine Users (N=53)	*p value vs Healthy Controls
Age (mean ± SD)	33.04 ± 10.93	45.87 ± 7.75	***<0.0001***
Female (%)	47.92%	35.85%	0.233
African American (%)	79.17%	88.68%	0.276
Years of Education (mean ± SD)	14.18 ± 2.26	12.35 ± 1.81	***<0.0001***
Smokers (%)	27.08%	88.10%	***<0.0001***
Marijuana Users (%)	14.58%	56.60%	***<0.0001***

*p < 0.05 versus healthy controls is bolded and italicized.

Recruitment site: Healthy Controls-University of Texas Health Science Center (n = 10), Virginia Commonwealth University (n=38); Cocaine Users-University of Texas Health Science Center (n = 10); Virginia Commonwealth University (n = 43).

**Table 2:** Correlational analyses of demographics with percent methylation of *HTR2A* promoter CpG sites.

Healthy Controls				
Characteristics	Site -1439	Site -1420	Site -1224	Site -253
Age (R Value, P value)	-0.086, 0.573	-0.111, 0.460	***-0.312, <0.05***	***-0.336, <0.05***
Years of Cocaine Use (R value, P Value)	–	–	–	–
Years of Smoking (R value, P Value)	–	–	–	–
**Cocaine Users**				
**Characteristics**	**Site -1439**	**Site -1420**	**Site -1224**	**Site -253**
Age (R Value, P value)	-0.078, 0.678	-0.249, 0.156	***-0.407, <0.05***	-0.269, 0.118
Years of Cocaine Use (R value, P Value)	0.035, 0.822	0.082, 0.587	0.039, 0.779	***-0.293, <0.05***
Years of Smoking (R value, P Value)	-0.102, 0.593	-0.045, 0.813	***-0.365, <0.05***	-0.171, 0.319

*p < 0.05 for correlation is bolded and italicized.

Within subject comparisons of healthy controls indicated a significant difference for percent methylation between the CpG sites [*χ*^2^(3) = 38.725; p < 0.05]. Dunn-Bonferroni multiple comparisons tests indicated significant differences for percent methylation at all sites (p < 0.05) *except* between sites -1224 and -1439 CpG as well as between sites -1420 and -253 for healthy controls. Within subject comparisons of cocaine users detected a significant difference for percent methylation between the CpG sites [*χ*^2^(3) = 27.98; p < 0.05]. Dunn-Bonferroni multiple comparisons tests indicated significant differences for percent methylation between sites -1224 and -1420 (*p* < 0.05) as well as between sites -1224 and -253 (*p* < 0.05) and no significant differences between any other CpG sites for cocaine users. Overall percent methylation between healthy controls and cocaine users was determined for each CpG site of interest (**Figures 1B–E**). There was no significant difference between healthy controls and cocaine users for CpG sites -1439 (n.s.; **Figure 1B**) and -1420 (n.s.; **Figure 1C**). Cocaine users displayed hypomethylation at CpG sites -1224 (p < 0.05, **Figure 1D**) and -253 (p < 0.05, **Figure 1E**) versus healthy controls.

### Levels of Impulsivity Correlate With *HTR2A* Promoter Percent Methylation at Site -1224 in Cocaine Users

**Figure 2:**
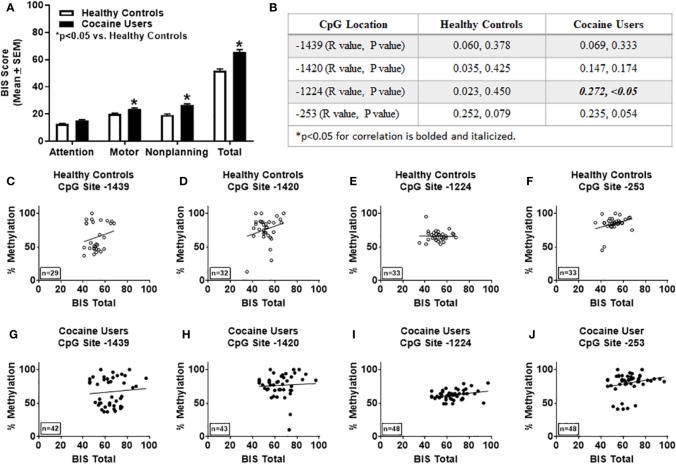
Levels of impulsivity correlate with percent methylation at site -1224 of the *HTR2A* promoter in cocaine users. **(A)** Impulsivity was determined using the Barratt Impulsiveness Scale (BIS-11). *p < 0.05 versus healthy controls. **(B)** Correlations and p values of percent methylation of the *HTR2A* promotor with total BIS scores at specific sites within the *HTR2A* promoter are represented in the table. Graphical representation of the correlations between percent methylation of the *HTR2A* promotor with total BIS-11 scores at specific sites within the *HTR2A* promoter for healthy controls **(C–F)** and cocaine users **(G–J)**. The number of individuals used in each correlation are indicated on the bottom left of the graph.

Overall levels of impulsivity were determined using the BIS-11 (2). Within subject analyses of healthy controls for BIS-11 (all measures) indicated that the assumption of sphericity was violated [*χ*^2^(5)=12.69; p < 0.05; Mauchly's test], therefore degrees of freedom were corrected using Huynh-Feldt estimates of sphericity (ϵ=0.895). The results show that there was a significant difference between measures of BIS-11 of the healthy controls, [F(2.69, 85.92)=776.10, p < 0.05]. Bonferroni correction for multiple comparisons showed significant difference between all measures of impulsivity (p < 0.05) except for BIS-11 motor vs BIS-11 non-planning for healthy controls. Within subject analyses of cocaine users for BIS-11 (all measures) indicated that the assumption of sphericity was violated [*χ*^2^(5)=48.88; p < 0.05; Mauchly's test], therefore degrees of freedom were corrected using Greenhouse-Geisser estimates of sphericity (ϵ=0.614). The results show that there was a significant difference between measures of BIS-11 of the cocaine users [F(1.84, 86.59)=1062.95, p < 0.05]. Bonferroni correction for multiple comparisons showed significant difference (p < 0.05) between all measures of the BIS-11 for cocaine users. Although levels of attention were not different between groups, cocaine users showed significantly higher scores on the BIS-11 for motor, non-planning, and total scores versus healthy controls (p < 0.05, **Figure 2A**). Total BIS-11 scores positively correlated with percent methylation at CpG site -1224 in cocaine users only (R=0.272, p < 0.05, **Figures 2B, I**). As noted above, cocaine users were significantly older than healthy controls (**Table 1**); after correcting for age, the correlation between total BIS-11 scores and percent methylation at CpG site -1224 trends toward significance (R=0.237, *p*=0.054). No significant correlations were detected between percent methylation at CpG sites -1439, -1420 or -235 and total BIS-11 scores in the healthy controls or cocaine users (n.s.; **Figures 2B–H, J**).

### Delay Discounting Rates Correlate With *HTR2A* Promotor Percent Methylation at Site -1420 in Healthy Controls

**Figure 3:**
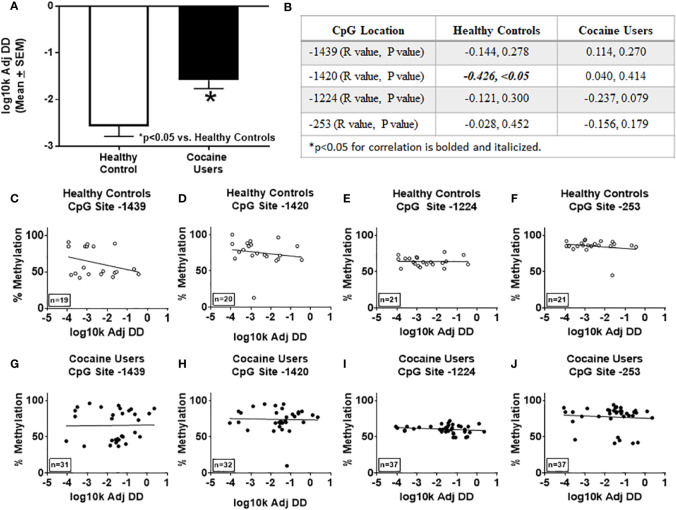
Delay discounting rates correlate with percent methylation at site -1420 of the *HTR2A* promotor in healthy controls. **(A)** Impulsive choice was determined using the delay discounting task in healthy controls and cocaine users. *p < 0.05 versus healthy controls. **(B)** Correlations and p values of percent methylation of the *HTR2A* promoter with the delay discounting rates at specific sites within the *HTR2A* promoter are represented in the table. Graphical representation of the correlations between percent methylation of the *HTR2A* promotor and delay discounting rates at specific sites within the *HTR2A* promoter for healthy controls **(C–F)** and cocaine users **(G–J)**. The number of individuals used in each correlation are indicated on the bottom left of the graph.

Using the delay discounting task to measure impulsive choice, a key facet of overall impulsivity (2, 6, 58, 59), correlational analyses between delay discounting scores and BIS-11 for healthy controls [total (R=0.1342, n.s.), attention (R=-0.1671, n.s.), motor (R=0.2233, n.s.), non-planning (R=0.1586, n.s.)] or cocaine users [total (R=-0.0239, n.s.), attention (R=0.0335, n.s.), motor (R=-0.1351, n.s.), non-planning (R=0.0229, n.s.)] were not significantly correlated. These data suggest that the BIS-11 and delay discounting task are independent measures of impulsivity (60). Cocaine users showed a significant preference for the smaller immediate reward over the larger delayed reward as compared to the healthy controls (p < 0.05, **Figure 3A**), corroborating previous findings (5). Discounting rates negatively correlated with percent methylation at CpG site -1420 in healthy controls only (R=0.4259, p < 0.05, **Figures 3B, D**), which remained significant after correcting for age (R=-0.504, p < 0.05). There were no significant correlations between percent methylation and the discounting scores at CpG sites -1439, -1224, or -253 in the healthy controls or cocaine users detected (n.s.; **Figures 3B, C, E–J**).

### Attentional Bias Correlates With *HTR2A* Promotor Percent Methylation at Site -253 in Cocaine Users

**Figure 4:**
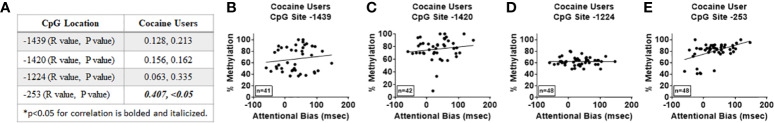
Attentional bias correlates with percent methylation at site -253 of the *HTR2A* promotor in cocaine users. Attentional bias was determined using the cocaine-word Stroop task in cocaine users. **(A)** Correlations and p values of percent methylation of the *HTR2A* promotor with mean reaction times (msec) at specific sites within the *HTR2A* promoter are represented in the table. **(B–E)** Graphical representation of the correlations between percent methylation of the *HTR2A* promotor and attentional bias at specific sites within the *HTR2A* promoter for cocaine users. The number of individuals used in each correlation are indicated on the bottom left of the graph.

Healthy controls (11.31 ± 8.8 sec) showed less attentional bias in the cocaine word Stroop task versus cocaine users (33.19 ± 6.6 sec; p < 0.05). Levels of attentional bias of cocaine users in the cocaine-word Stroop task positively correlated with percent methylation at CpG site -253 (R=0.4065, p < 0.05, **Figures 4A, E**) and remained significant after correcting for age and years of cocaine use (R=0.410, p < 0.05). There were no significant correlations observed between percent methylation and attentional bias at CpG sites -1439, -1420, or -1224 in cocaine users (n.s.; **Figures 4A–D**).

## Discussion

We discovered in the present study that cocaine users exhibited higher levels of impulsivity as measured by the BIS-11 and delay discounting task, as expected. Cocaine users exhibited hypomethylation at CpG sites -1224 and -253 but not at CpG sites -1439 and -1420 versus healthy controls. The percent methylation of CpG site -1224, but not -1439, -1420, or -235, of the *HTR2A* promoter trended towards a positive correlation with total BIS-11 scores in cocaine users. A negative correlation between delay discounting rates and percent methylation at site -1420 was observed in healthy controls. In addition, levels of attentional bias positively correlated with percent methylation of CpG site -253, but not other sites, in cocaine users. We also determined that the rs6311 SNP was present equally in the healthy controls and cocaine users. Taken together, this study provides evidence that individual differences in CUD relapse-related behaviors associate with the pattern of methylation within the *HTR2A* promoter.

Cytosine methylation patterns vary substantially across different cells of the same organism, and these patterns can change over time within the same cell. Of note, the association between methylation at site -1224 and total BIS-11 levels trended towards significance after correction for age, suggesting age may have a small impact on the association. Chronological age has been shown to influence DNA methylation in lymphocytes, with increasing age typically associated with DNA hypomethylation (61, 62). The CpG site -1224, which positively correlated with total impulsivity levels in cocaine users, is found in the Sp1 transcription factor binding site (52, 54). Interestingly, methylation of Sp1 binding sites increases with cocaine exposure (63), which results in reduced Sp1 binding (64) and decreased transcription of its target genes (63). Thus, the regulatory role of this CpG site over the Sp1 transcription factor is an excellent future candidate for determining gene-environment interactions for cocaine-dependent individuals with high trait impulsivity.

Interestingly, the pattern of methylation detected herein differed between the two measures of impulsivity and is most likely a reflection of the tools employed, that is a subjective (BIS-11) versus objective (delay discounting) measure. The BIS-11 is a onetime questionnaire designed to elicit individual reporting of their past acts of impulsiveness (2). In contrast, the delay discounting task is an active measure of impulsive choice or the tendency to prefer smaller, immediate rewards over larger, delayed rewards (2, 6, 58, 59). Results from the delay discounting task indicated a distinct pattern of *HTR2A* promoter methylation as compared to the BIS-11. A negative correlation between methylation at the CpG site -1420 and delay discounting rates for healthy controls, even after correcting for age, was detected, suggesting the pattern of methylation at CpG site -1420 could be disrupted in cocaine users with high impulsive choice. The CpG site -1420 for the *HTR2A* is found in the binding site for the GR and PR (52, 57). Glucocorticoids and progesterone levels are elevated after administration of cocaine (65, 66) resulting in increased progesterone receptor DNA binding following acute cocaine injection (66). Further, genetic removal or pharmacological inhibition of the GR results in reduced cocaine self-administration in rodents (67). Thus, we hypothesize that dysregulation of methylation in high impulsive choice cocaine users versus healthy controls at site -1420 could be the result of altered glucocorticoid and progesterone levels associated with cocaine use (65, 66) resulting in increased receptor DNA binding (66). Future studies are warranted to explore the hypothesis that specific types of impulsivity (e.g., impulsive action, impulsive choice) may present with their own unique gene-environment interactions.

Attentional bias in cocaine users, i.e. cocaine cue reactivity, positively associated with methylation at CpG site -253 within a known silencer region of the *HTR2A* promoter (63). In general, methylation of silencers prevents repressor proteins from binding and potentially abolish their repressive function on gene transcription (Jones, 2001). Removal of the *HTR2A* silencer region robustly increased promoter activity as determined by reporter assays *in vitro* (54). Broadly, these data align with preclinical studies reporting a putative hyperfunctional 5-HT_2A_R system in relapse-related behaviors (for reviews) (6, 7). Studies to investigate the causal relationship between epigenetic reprogramming of the peripherally and centrally localized 5-HT_2A_R and CUD relapse-like related behaviors are warranted.

The rs6311 SNP results in the loss of the -1439 CpG site and introduces a new transcription factor binding site for E47 (52, 53). E47 is a member of the helix-loop-helix transcription factor family which upon binding can result in increased promoter activity of the *HTR2A* (68). Further, this family of transcription factors play a critical role in corticogenesis (69). The rs6311 SNP also associates with levels of disease severity (70), drug response (71), and is found at higher frequencies in schizophrenic patients (72). The rs6311 SNP also associates with early onset obsessive-compulsive disorder (73) and impaired impulse control in individuals diagnosed with schizophrenia (73). While this SNP actually removes the -1439 CpG island, impacts transcription levels of the *HTR2A* gene and is associated with a number of other neuropsychiatric disorders, we did not identify a bias for cocaine users to express this SNP to a greater degree than healthy controls. The prevalence of the rs6311 SNP is approximately 44% in the global population (1,000 Genomes). However, we may have had too small a sample size to detect allelic differences between the healthy controls and cocaine users.

Limitations of this study are in the sample size and the need to replicate these findings in additional cohorts measuring the same behaviors of impulsivity (both subjective and objective) and attentional bias. Furthermore, while our targeted gene and transcription factor binding profile is key to implicating molecular regulation of the *HTR2A* in relapse-related behaviors in cocaine-dependent individuals, it is wholly conceivable that an unidentified gene by environment interaction and/or the highly polygenic nature of psychiatric disorders exists, such that the methylation profile of the *HTR2A* may aggregate with an unidentified genetic/epigenetic target to contribute to phenotypic variation (74, 75). Further, a major part of epigenetic regulation is the impact on gene and protein levels. There is evidence that methylation of the *HTR2A* promoter associates with levels of *HTR2A* mRNA expression in postmortem brain (21, 36), suggesting that changes in methylation may influence expression of the *HTR2A* gene and ultimately protein levels. Finally, lymphocytes have been used to demonstrate aberrant global and/or site-specific DNA methylation in several psychiatric disorders (e.g., bipolar disorder, schizophrenia) (76, 77) justifying our initial approach to measure DNA methylation in blood of individuals characterized for clinical and behavioral impulsivity or cocaine cue reactivity. DNA methylation in the peripheral blood cells of several neuropsychiatric disorders have also been shown to mirror changes in the brain (78–82). Additionally, there is evidence that percent methylation of the *HTR2A* promoter from human peripheral leukocytes associates with methylation from the human temporal cortex (36). This suggests that peripheral blood lymphocytes can be used as marker of neuropsychiatric disorders, even where the direct cause to neural changes in the brain is, yet, unclear. Thus, future studies are required to compare DNA methylation in blood versus brain ideally in humans, although there is an obviously significant barrier to this research. We can, however, compare blood and brain global and site-specific DNA methylation patterns in well-controlled rodent studies.

The discovery and validation of biological and phenotypic individual differences in relapse-related behavioral vulnerability would greatly improve objective risk assessment of disease progress, predict response to treatments, and subgroup patients to receive a more optimized treatment regimen for this multifaceted disease (for review) (83). Currently, a key tool used to predict patient response to treatment is the cocaine selective severity assessment (CSSA), which is an 18-item questionnaire that measures levels of cocaine withdrawal (84–86). The CSSA does not account for the complex nature of CUD with its interlocking phenotypes of impulsivity and cocaine cue reactivity. Other measures such as neuroimaging, metabolomics, transcriptomics, genetics, and epigenetics could be used to supplement the current CSSA questionnaire and further stratify cocaine-dependent individuals into specific treatment subgroups. As DNA methylation is a memory of past gene-environment interactions, consideration of individual differences in targeted gene methylation profiles might allow identification of those individuals who have the highest risk for relapse-related behaviors, and subsequently lead to rational behavioral or pharmacotherapeutic strategies for minimizing damage where abstinence is not successful.

## Data Availability Statement

The sequencing data of site -1438 (rs6311) presented in this study is publicly available and can be found here: https://www.ncbi.nlm.nih.gov/clinvar/, SCV001142620. The remaining raw data supporting the conclusions of this article will be made available by the authors, without undue reservation, to all qualified researchers.

## Ethics Statement

The studies involving human participants were reviewed and approved by the Institutional Review Boards at the University of Texas Health Science Center at Houston, Virginia Commonwealth University, and the University of Texas Medical Branch. The patients/participants provided their written informed consent to participate in this study.

## Author Contributions

ML performed the data analyses and drafted the manuscript. DR performed behavioral assessments. AM and RP designed and performed the pyrosequencing experiments. KC, FM, and NA conceptualized the project, oversaw experimental design/interpretation/analyses, and wrote/edited the manuscript.

## Funding

This work was supported by the National Institute on Drug Abuse grants P50 DA033935 (KC, FM, NA), and T32 DA007287 (ML).

## Conflict of Interest

FM has current research funding from Indivior Pharmaceuticals and Nektar Therapeutics for research unrelated to this study.

The remaining authors declare that the research was conducted in the absence of any commercial or financial relationships that could be construed as a potential conflict of interest.

## References

[1] McCall Jones C Baldwin GT Compton WM Recent Increases in Cocaine-Related Overdose Deaths and the Role of Opioids. Am J Public Health (2017) 107(3):430–2. 10.2105/ajph.2016.303627 PMC5296707 28177817

[2] Moeller FG Barratt ES Dougherty DM Schmitz JM Swann AC Psychiatric aspects of impulsivity. Am J Psychiatry (2001) 158(11):1783–93. 10.1176/appi.ajp.158.11.1783 11691682

[3] Moeller FG Barratt ES Fischer CJ Dougherty DM Reilly EL Mathias CW P300 event-related potential amplitude and impulsivity in cocaine-dependent subjects. Neuropsychobiology (2004) 50(2):167–73. 10.1159/000079110 15292673

[4] Moeller FG Dougherty DM Barratt ES Oderinde V Mathias CW Harper RA Increased impulsivity in cocaine dependent subjects independent of antisocial personality disorder and aggression. Drug Alcohol Depend (2002) 68(1):105–11. 10.1016/S0376-8716(02)00106-0 12167556

[5] Coffey SF Gudleski GD Saladin ME Brady KT Impulsivity and rapid discounting of delayed hypothetical rewards in cocaine-dependent individuals. Exp Clin Psychopharmacol (2003) 11(1):18–25. 10.1037/1064-1297.11.1.18 12622340

[6] Cunningham KA Anastasio NC Serotonin at the nexus of impulsivity and cue reactivity in cocaine addiction. Neuropharmacology (2014) 76 Pt B:460–78. 10.1016/j.neuropharm.2013.06.030 PMC4090081 23850573

[7] Howell LL Cunningham KA Serotonin 5-HT2 receptor interactions with dopamine function: implications for therapeutics in cocaine use disorder. Pharmacol Rev (2015) 67(1):176–97. 10.1124/pr.114.009514 PMC4279075 25505168

[8] Carter BL Tiffany ST Meta-analysis of cue-reactivity in addiction research. Addiction (1999) 94(3):327–40. 10.1046/j.1360-0443.1999.9433273.x 10605857

[9] Field M Cox WM Attentional bias in addictive behaviors: a review of its development, causes, and consequences. Drug Alcohol Depend (2008) 97(1-2):1–20. 10.1016/j.drugalcdep.2008.03.030 18479844

[10] Moeller FG Dougherty DM Barratt ES Schmitz JM Swann AC Grabowski J The impact of impulsivity on cocaine use and retention in treatment. JSubstAbuse Treat (2001) 21(4):193–8. 10.1016/S0740-5472(01)00202-1 11777668

[11] O'Brien CP Childress AR Ehrman R Robbins SJ Conditioning factors in drug abuse: can they explain compulsion? J Psychopharmacol (1998) 12(1):15–22. 10.1177/026988119801200103 9584964

[12] Carter BL Tiffany ST Cue-reactivity and the future of addiction research. Addiction (1999) 94(3):349–51. 10.1046/j.1360-0443.1999.9433273.x 10605863

[13] Modesto-Lowe V Kranzler HR Using cue reactivity to evaluate medications for treatment of cocaine dependence: a critical review. Addiction (1999) 94(11):1639–51. 10.1046/j.1360-0443.1999.941116393.x 10892004

[14] Patkar AA Murray HW Mannelli P Gottheil E Weinstein SP Vergare MJ Pre-treatment measures of impulsivity, aggression and sensation seeking are associated with treatment outcome for African-American cocaine-dependent patients. JAddictDis (2004) 23(2):109–22. 10.1300/J069v23n02_08 15132346

[15] Abdolmaleky HM Smith CL Faraone SV Shafa R Stone W Glatt SJ Methylomics in psychiatry: Modulation of gene-environment interactions may be through DNA methylation. AmJMedGenetB NeuropsychiatrGenet (2004) 127B(1):51–9. 10.1002/ajmg.b.20142 15108180

[16] Reik W Stability and flexibility of epigenetic gene regulation in mammalian development. Nature (2007) 447(7143):425–32. 10.1038/nature05918 17522676

[17] Lv J Xin Y Zhou W Qiu Z The epigenetic switches for neural development and psychiatric disorders. J Genet Genomics (2013) 40(7):339–46. 10.1016/j.jgg.2013.04.007 23876774

[18] Bird A DNA methylation patterns and epigenetic memory. Genes Dev (2002) 16(1):6–21. 10.1101/gad.947102 11782440

[19] Huang B Li G Jiang XH Fate determination in mesenchymal stem cells: a perspective from histone-modifying enzymes. Stem Cell Res Ther (2015) 6:35. 10.1186/s13287-015-0018-0 25890062 PMC4365520

[20] Miranda TB Jones PA DNA methylation: the nuts and bolts of repression. J Cell Physiol (2007) 213(2):384–90. 10.1002/jcp.21224 17708532

[21] Cheah SY Lawford BR Young RM Morris CP Voisey J mRNA Expression and DNA Methylation Analysis of Serotonin Receptor 2A (HTR2A) in the Human Schizophrenic Brain. Genes (Basel) (2017) 8(1):1–11. 10.3390/genes8010014 PMC5295009 28054990

[22] Grayson DR Guidotti A The dynamics of DNA methylation in schizophrenia and related psychiatric disorders. Neuropsychopharmacology (2013) 38(1):138–66. 10.1038/npp.2012.125 PMC3521968 22948975

[23] Robison AJ Nestler EJ Transcriptional and epigenetic mechanisms of addiction. Nat Rev Neurosci (2011) 12(11):623–37. 10.1038/nrn3111 PMC3272277 21989194

[24] Anastasio NC Stoffel EC Fox RG Bubar MJ Rice KC Moeller FG Serotonin (5-hydroxytryptamine) 5-HT_2A_ receptor: Association with inherent and cocaine-evoked behavioral disinhibition in rats. BehavPharmacol (2011) 22(3):248–61. 10.1097/FBP.0b013e328345f90d PMC3124821 21499079

[25] Fletcher PJ Tampakeras M Sinyard J Higgins GA Opposing effects of 5-HT(2A) and 5-HT(2C) receptor antagonists in the rat and mouse on premature responding in the five-choice serial reaction time test. Psychopharmacol (Berl) (2007) 195(2):223–34. 10.1007/s00213-007-0891-z 17673981

[26] Winstanley CA Theobald DE Dalley JW Glennon JC Robbins TW 5-HT2A and 5-HT2C receptor antagonists have opposing effects on a measure of impulsivity: interactions with global 5-HT depletion. Psychopharmacol (Berl) (2004) 176(3-4):376–85. 10.1007/s00213-004-1884-9 15232674

[27] Fletcher PJ Grottick AJ Higgins GA Differential effects of the 5-HT2A receptor antagonist M100,907 and the 5-HT2C receptor antagonist SB242,084 on cocaine-induced locomotor activity, cocaine self-administration and cocaine-induced reinstatement of responding. Neuropsychopharmacology (2002) 27(4):576–86. 10.1016/S0893-133X(02)00342-1 12377394

[28] Nic Dhonnchadha BA Fox RG Stutz SJ Rice KC Cunningham KA Blockade of the serotonin 5-HT2A receptor suppresses cue-evoked reinstatement of cocaine-seeking behavior in a rat self-administration model. Behav Neurosci (2009) 123(2):382–96. 10.1037/a0014592 PMC3830454 19331461

[29] Sholler DJ Stutz SJ Fox RG Boone EL Wang Q Rice KC The 5-HT2A Receptor (5-HT2AR) Regulates Impulsive Action and Cocaine Cue Reactivity in Male Sprague-Dawley Rats. J Pharmacol Exp Ther (2019) 368(1):41–9. 10.1124/jpet.118.251199 PMC6290084 30373886

[30] Hadamitzky M Koch M Effects of acute intra-cerebral administration of the 5-HT(2A/C) receptor ligands DOI and ketanserin on impulse control in rats. BehavBrain Res (2009) 204(1):88–92. 10.1016/j.bbr.2009.05.021 19467270

[31] Blasio A Narayan AR Kaminski BJ Steardo L Sabino V Cottone P A modified adjusting delay task to assess impulsive choice between isocaloric reinforcers in non-deprived male rats: effects of 5-HT(2A/C) and 5-HT (1A) receptor agonists. Psychopharmacol (Berl) (2012) 219(2):377–86. 10.1007/s00213-011-2517-8 PMC3936353 21989803

[32] Persons AL Tedford SE Celeste T Mirtazapine and ketanserin alter preference for gambling-like schedules of reinforcement in rats. Prog Neuropsychopharmacol Biol Psychiatry (2017) 77:178–84. 10.1016/j.pnpbp.2017.03.027 PMC5656013 28412411

[33] Ishii K Matsunaga M Noguchi Y Yamasue H Ochi M Ohtsubo Y A polymorphism of serotonin 2A receptor (5-HT2AR) influences delay discounting. Pers Individ Dif (2018) 121:193–9. 10.1016/j.paid.2017.03.011

[34] Abdolmaleky HM Zhou JR Thiagalingam S Smith CL Epigenetic and pharmacoepigenomic studies of major psychoses and potentials for therapeutics. Pharmacogenomics (2008) 9(12):1809–23. 10.2217/14622416.9.12.1809 19072640

[35] Bunzel R Blumcke I Cichon S Normann S Schramm J Propping P Polymorphic imprinting of the serotonin-2A (5-HT2A) receptor gene in human adult brain. Brain ResMolBrain Res (1998) 59(1):90–2. 10.1016/S0169-328X(98)00146-6 9729300

[36] Polesskaya OO Aston C Sokolov BP Allele C-specific methylation of the 5-HT2A receptor gene: evidence for correlation with its expression and expression of DNA methylase DNMT1. J Neurosci Res (2006) 83(3):362–73. 10.1002/jnr.20732 16358338

[37] First MB Structured Clinical Interview for DSM-IV Axis I Disorders. New York: Biometrics Research Department (1997).

[38] Liu S Lane SD Schmitz JM Waters AJ Cunningham KA Moeller FG Relationship between attentional bias to cocaine-related stimuli and impulsivity in cocaine-dependent subjects. Am J Drug Alcohol Abuse (2011) 37(2):117–22. 10.3109/00952990.2010.543204 PMC3110662 21204739

[39] Ahn WY Ramesh D Moeller FG Vassileva J Utility of Machine-Learning Approaches to Identify Behavioral Markers for Substance Use Disorders: Impulsivity Dimensions as Predictors of Current Cocaine Dependence. Front Psychiatry (2016) 7:34. 10.3389/fpsyt.2016.00034 27014100 PMC4785183

[40] Ma L Steinberg JL Cunningham KA Lane SD Bjork JM Neelakantan H Inhibitory behavioral control: A stochastic dynamic causal modeling study comparing cocaine dependent subjects and controls. NeuroImage Clin (2015) 7:837–47. 10.1016/j.nicl.2015.03.015 PMC4459041 26082893

[41] Bloom EL Matsko SV Cimino CR The relationship between cigarette smoking and impulsivity: A review of personality, behavioral, and neurobiological assessment. Addict Res Theory (2014) 22(5):386–97. 10.3109/16066359.2013.867432

[42] Patton JH Stanford MS Barratt ES Factor structure of the Barratt impulsiveness scale. JClinPsychol (1995) 51(6):768–74. 10.1002/1097-4679(199511)51:6<768::AID-JCLP2270510607>3.0.CO;2-1 8778124

[43] Johnson MW Bickel WK Within-subject comparison of real and hypothetical money rewards in delay discounting. J Exp Anal Behav (2002) 77(2):129–46. 10.1901/jeab.2002.77-129 PMC1284852 11936247

[44] Heil SH Johnson MW Higgins ST Bickel WK Delay discounting in currently using and currently abstinent cocaine-dependent outpatients and non-drug-using matched controls. Addict Behav (2006) 31(7):1290–4. 10.1016/j.addbeh.2005.09.005 16236455

[45] Mazur JE Coe D Tests of transitivity in choices between fixed and variable reinforcer delays. J Exp Anal Behav (1987) 47(3):287–97. 10.1901/jeab.1987.47-287 PMC1348312 3612019

[46] Hester R Dixon V Garavan H A consistent attentional bias for drug-related material in active cocaine users across word and picture versions of the emotional Stroop task. Drug Alcohol Depend (2006) 81(3):251–7. 10.1016/j.drugalcdep.2005.07.002 16095852

[47] Liu S Lane SD Schmitz JM Green CE Cunningham KA Moeller FG Increased intra-individual reaction time variability in cocaine-dependent subjects: role of cocaine-related cues. AddictBehav (2012) 37(2):193–7. 10.1016/j.addbeh.2011.10.003 PMC3253315 22047976

[48] Anastasio NC Liu S Maili L Swinford SE Lane SD Fox RG Variation within the serotonin (5-HT) 5-HT(2)C receptor system aligns with vulnerability to cocaine cue reactivity. Trans Psychiatry (2014) 4:e369. 10.1038/tp.2013.131 PMC3966037 24618688

[49] Field M Munafo MR Franken IH A meta-analytic investigation of the relationship between attentional bias and subjective craving in substance abuse. PsycholBull (2009) 135(4):589–607. 10.1037/a0015843 PMC2999821 19586163

[50] Ma L Steinberg JL Cunningham KA Bjork JM Lane SD Schmitz JM Altered anterior cingulate cortex to hippocampus effective connectivity in response to drug cues in men with cocaine use disorder. Psychiatry Res Neuroimaging (2018) 271:59–66. 10.1016/j.pscychresns.2017.10.012 29108734 PMC5741507

[51] Cox WM Fadardi JS Pothos EM The addiction-stroop test: Theoretical considerations and procedural recommendations. PsycholBull (2006) 132(3):443–76. 10.1037/0033-2909.132.3.443 16719569

[52] Falkenberg VR Gurbaxani BM Unger ER Rajeevan MS Functional genomics of serotonin receptor 2A (HTR2A): interaction of polymorphism, methylation, expression and disease association. Neuromol Med (2011) 13(1):66–76. 10.1007/s12017-010-8138-2 PMC3044825 20941551

[53] Smith AK Dimulescu I Falkenberg VR Narasimhan S Heim C Vernon SD Genetic evaluation of the serotonergic system in chronic fatigue syndrome. Psychoneuroendocrinology (2008) 33(2):188–97. 10.1016/j.psyneuen.2007.11.001 18079067

[54] Zhu QS Chen K Shih JC Characterization of the human 5-HT2A receptor gene promoter. J Neurosci (1995) 15(7 Pt 1):4885–95. 10.1523/JNEUROSCI.15-07-04885.1995 PMC6577879 7623119

[55] Bolwig GM Hearing P Interaction of nuclear factor EF-1A with the polyomavirus enhancer region. J Virol (1991) 65(4):1884–92. 10.1128/JVI.65.4.1884-1892.1991 PMC240000 1848308

[56] Higashino F Yoshida K Fujinaga Y Kamio K Fujinaga K Isolation of a cDNA encoding the adenovirus E1A enhancer binding protein: a new human member of the ets oncogene family. Nucleic Acids Res (1993) 21(3):547–53. 10.1093/nar/21.3.547 PMC309151 8441666

[57] Falkenberg VR Rajeevan MS Identification of a potential molecular link between the glucocorticoid and serotonergic signaling systems. J Mol Neurosci (2010) 41(2):322–7. 10.1007/s12031-009-9320-6 20052562

[58] Weafer J Baggott MJ de Wit H Test-retest reliability of behavioral measures of impulsive choice, impulsive action, and inattention. Exp Clin Psychopharmacol (2013) 21(6):475–81. 10.1037/a0033659 PMC4266373 24099351

[59] Hamilton KR Mitchell MR Wing VC Balodis IM Bickel WK Fillmore M Choice impulsivity: Definitions, measurement issues, and clinical implications. Pers Disord (2015) 6(2):182–98. 10.1037/per0000099 PMC4535726 25867841

[60] Duckworth AL Kern ML A Meta-Analysis of the Convergent Validity of Self-Control Measures. J Res Pers (2011) 45(3):259–68. 10.1016/j.jrp.2011.02.004 PMC3105910 21643479

[61] Delgado-Morales R Agis-Balboa RC Esteller M Berdasco M Epigenetic mechanisms during ageing and neurogenesis as novel therapeutic avenues in human brain disorders. Clin Epigenet (2017) 9:67. 10.1186/s13148-017-0365-z PMC5493012 28670349

[62] Horvath S Raj K DNA methylation-based biomarkers and the epigenetic clock theory of ageing. Nat Rev Genet (2018) 19(6):371–84. 10.1038/s41576-018-0004-3 29643443

[63] Meyer K Zhang H Zhang L Direct effect of cocaine on epigenetic regulation of PKCepsilon gene repression in the fetal rat heart. J Mol Cell Cardiol (2009) 47(4):504–11. 10.1016/j.yjmcc.2009.06.004 PMC2739252 19538969

[64] Clark SJ Harrison J Molloy PL Sp1 binding is inhibited by (m)Cp(m)CpG methylation. Gene (1997) 195(1):67–71. 10.1016/S0378-1119(97)00164-9 9300822

[65] Mello NK Mendelson JH Cocaine's effects on neuroendocrine systems: Clinical and preclinical studies. Pharmacol Biochem Behav (1997) 57(3):571–99. 10.1016/S0091-3057(96)00433-9 9218281

[66] Wu HB Fabian S Jenab S Quinones-Jenab V Progesterone receptors activation after acute cocaine administration. Brain Res (2006) 1126(1):188–92. 10.1016/j.brainres.2006.09.074 17109827

[67] Deroche-Gamonet V Sillaber I Aouizerate B Izawa R Jaber M Ghozland S The glucocorticoid receptor as a potential target to reduce cocaine abuse. J Neurosci (2003) 23(11):4785–90. 10.1523/JNEUROSCI.23-11-04785.2003 PMC6740779 12805318

[68] Parsons MJ D'Souza UM Arranz MJ Kerwin RW Makoff AJ The -1438A/G polymorphism in the 5-hydroxytryptamine type 2A receptor gene affects promoter activity. Biol Psychiatry (2004) 56(6):406–10. 10.1016/j.biopsych.2004.06.020 15364038

[69] Ross SE Greenberg ME Stiles CD Basic helix-loop-helix factors in cortical development. Neuron (2003) 39(1):13–25. 10.1016/S0896-6273(03)00365-9 12848929

[70] Quednow BB Kuhn KU Mossner R Schwab SG Schuhmacher A Maier W Sensorimotor gating of schizophrenia patients is influenced by 5-HT2A receptor polymorphisms. Biol Psychiatry (2008) 64(5):434–7. 10.1016/j.biopsych.2008.02.019 18420180

[71] Penas-Lledo EM Dorado P Caceres MC de la Rubia A Llerena A Association between T102C and A-1438G polymorphisms in the serotonin receptor 2A (5-HT2A) gene and schizophrenia: relevance for treatment with antipsychotic drugs. Clin Chem Lab Med (2007) 45(7):835–8. 10.1515/CCLM.2007.181 17617023

[72] Chen SF Shen YC Chen CH HTR2A A-1438G/T102C polymorphisms predict negative symptoms performance upon aripiprazole treatment in schizophrenic patients. Psychopharmacol (Berl) (2009) 205(2):285–92. 10.1007/s00213-009-1538-z 19387614

[73] Walitza S Bove DS Romanos M Renner T Held L Simons M Pilot study on HTR2A promoter polymorphism, -1438G/A (rs6311) and a nearby copy number variation showed association with onset and severity in early onset obsessive-compulsive disorder. J Neural Transm (Vienna) (2012) 119(4):507–15. 10.1007/s00702-011-0699-1 21874579

[74] Gray JC MacKillop J Weafer J Hernandez KM Gao J Palmer AA Genetic analysis of impulsive personality traits: Examination of a priori candidates and genome-wide variation. Psychiatry Res (2018) 259:398–404. 10.1016/j.psychres.2017.10.047 29120849 PMC5742029

[75] Sanchez-Roige S Fontanillas P Elson SL Gray JC de Wit H MacKillop J Genome-Wide Association Studies of Impulsive Personality Traits (BIS-11 and UPPS-P) and Drug Experimentation in up to 22,861 Adult Research Participants Identify Loci in the CACNA1I and CADM2 genes. J Neurosci (2019) 39(13):2562–72. 10.1523/JNEUROSCI.2662-18.2019 PMC6435820 30718321

[76] Kuratomi G Iwamoto K Bundo M Kusumi I Kato N Iwata N Aberrant DNA methylation associated with bipolar disorder identified from discordant monozygotic twins. MolPsychiatry (2008) 13(4):429–41. 10.1038/sj.mp.4002001 17471289

[77] Tsujita T Niikawa N Yamashita H Imamura A Hamada A Nakane Y Genomic discordance between monozygotic twins discordant for schizophrenia. AmJPsychiatry (1998) 155(3):422–4. 10.1176/ajp.155.3.422 9501757

[78] Wang SC Oelze B Schumacher A Age-specific epigenetic drift in late-onset Alzheimer's disease. PloS One (2008) 3(7):e2698. 10.1371/journal.pone.0002698 18628954 PMC2444024

[79] Walton E Hass J Liu J Roffman JL Bernardoni F Roessner V Correspondence of DNA Methylation Between Blood and Brain Tissue and Its Application to Schizophrenia Research. Schizophr Bull (2016) 42(2):406–14. 10.1093/schbul/sbv074 PMC4753587 26056378

[80] Abdolmaleky HM Nohesara S Ghadirivasfi M Lambert AW Ahmadkhaniha H Ozturk S DNA hypermethylation of serotonin transporter gene promoter in drug naive patients with schizophrenia. Schizophr Res (2014) 152(2-3):373–80. 10.1016/j.schres.2013.12.007 PMC7863587 24411530

[81] Guidotti A Auta J Davis JM Dong E Gavin DP Grayson DR Toward the identification of peripheral epigenetic biomarkers of schizophrenia. J Neurogenet (2014) 28(1-2):41–52. 10.3109/01677063.2014.892485 24702539 PMC4112595

[82] Nguyen A Rauch TA Pfeifer GP Hu VW Global methylation profiling of lymphoblastoid cell lines reveals epigenetic contributions to autism spectrum disorders and a novel autism candidate gene, RORA, whose protein product is reduced in autistic brain. FASEB J (2010) 24(8):3036–51. 10.1096/fj.10-154484 PMC2909294 20375269

[83] Bough KJ Amur S Lao G Hemby SE Tannu NS Kampman KM Biomarkers for the development of new medications for cocaine dependence. Neuropsychopharmacology (2014) 39(1):202–19. 10.1038/npp.2013.210 PMC3857653 23979119

[84] Gawin FH Kleber HD Abstinence symptomatology and psychiatric diagnosis in cocaine abusers. Clinical observations. ArchGenPsychiatry (1986) 43(2):107–13. 10.1001/archpsyc.1986.01800020013003 3947206

[85] Kampman KM Volpicelli JR Mulvaney F Rukstalis M Alterman AI Pettinati H Cocaine withdrawal severity and urine toxicology results from treatment entry predict outcome in medication trials for cocaine dependence. AddictBehav (2002) 27(2):251–60. 10.1016/S0306-4603(01)00171-X 11817766

[86] Mulvaney FD Alterman AI Boardman CR Kampman K Cocaine abstinence symptomatology and treatment attrition. J Subst Abuse Treat (1999) 16(2):129–35. 10.1016/S0740-5472(98)00017-8 10023610

